# Supplementary material for: Development of a computer-aided design software for the quantitative evaluation of aesthetic damage
Source: PLoS One. 2019 Dec 18;14(12):e0226322. doi: 10.1371/journal.pone.0226322 (PMC6919621; doi:10.1371/journal.pone.0226322)
Supplement: S1 Code site — 1. (ZIP) [file pone.0226322.s001.zip › [EESC_jr][Projeto]Relatorio_07_21/vendors/select2/docs/_includes/options-old/adapters.html]

# Adapters

Select2 allows plugins to add additional functionality through the core
adapters. You can change almost anything involving the way Select2 works
to the way Select2 interacts with the page by modifying the core adapters.
Most third-party plugins should provide decorators (used to wrap adapters)
and custom adapters that you can use.

Each adapter contains a set of methods which will must always be defined.
Along with the global methods that all adapters must implement, these
methods must be implemented.

## All adapters

All adapters must implement a set of methods that Select2 will use to
display them and bind any internal events.

```
// The basic HTML that should be rendered by Select2. A jQuery or DOM element
// should be returned, which will automatically be placed by Select2 within the
// DOM.
//
// @returns A jQuery or DOM element that contains any elements that must be
//   rendered by Select2.
Adapter.render = function () {
  return $jq;
};

// Bind to any Select2 or DOM events.
//
// @param container The Select2 object that is bound to the jQuery element.  You
//   can listen to Select2 events with `on` and trigger Select2 events using the
//   `trigger` method.
// @param $container The jQuery DOM node that all default adapters will be
//   rendered within.
Adapter.bind = function (container, $container) { };

// Position the DOM element within the Select2 DOM container, or in another
// place. This allows adapters to be located outside of the Select2 DOM,
// such as at the end of the document or in a specific place within the Select2
// DOM node.
//
// Note: This method is not called on data adapters.
//
// @param $rendered The rendered DOM element that was returned from the call to
//   `render`. This may have been modified by Select2, but the root element
//   will always be the same.
// @param $defaultContainer The default container that Select2 will typically
//   place the rendered DOM element within. For most adapters, this is the
//   Select2 DOM element.
Adapter.position = function ($rendered, $defaultContainer) { };

// Destroy any events or DOM elements that have been created.
// This is called when `select2("destroy")` is called on an element.
Adapter.destroy = function () { };
```

## Container (selection)

The selection is what is shown to the user as a replacement of the
standard `<select>` box. It controls the display of the
selection option(s), as well anything else that needs to be embedded
within the container, such as a search box.

Key
:   `selectionAdapter`

Default
:   `SingleSelection` or
    `MultipleSelection`

Base
:   `BaseSelection`

```
// Update the selected data.
//
// @param data An array of data objects that have been generated by the data
//   adapter. If no objects should be selected, an empty array will be passed.
//
// Note: An array will always be passed into this method, even if Select2 is
// attached to a source which only accepts a single selection.
SelectionAdapter.update = function (data) { };
```

## Data set

The data set is what Select2 uses to generate the possible results that
can be selected, as well as the currently selected results.

Key
:   `dataAdapter`

Default
:   `SelectAdapter`

Base
:   `BaseAdapter`

```
// Get the currently selected options. This is called when trying to get the
// initial selection for Select2, as well as when Select2 needs to determine
// what options within the results are selected.
//
// @param callback A function that should be called when the current selection
//   has been retrieved. The first parameter to the function should be an array
//   of data objects.
DataAdapter.current = function (callback) {
  callback(currentData);
}

// Get a set of options that are filtered based on the parameters that have
// been passed on in.
//
// @param params An object containing any number of parameters that the query
//   could be affected by. Only the core parameters will be documented.
// @param params.term A user-supplied term. This is typically the value of the
//   search box, if one exists, but can also be an empty string or null value.
// @param params.page The specific page that should be loaded. This is typically
//   provided when working with remote data sets, which rely on pagination to
//   determine what objects should be displayed.
// @param callback The function that should be called with the queried results.
DataAdapter.query = function (params, callback) {
  callback(queryiedData);
}
```

## Dropdown

The dropdown adapter defines the main container that the dropdown should
be held in. **It does not define any extra methods that can be used
for decorators**, but it is common for decorators to attach to the
`render` and `position` methods to alter how the
dropdown is altered and positioned.

Key
:   `dropdownAdapter`

Default
:   `DropdownAdapter`

## Results

The results adapter controls the list of results that the user can select
from. While the results adapter does not define any additional methods
that must be implemented, it makes extensive use of the Select2 event
system for controlling the display of results and messages.

Key
:   `resultsAdapter`

Default
:   `ResultsAdapter`
